# Regulation of tissue factor activity by interaction with the first PDZ domain of MAGI1

**DOI:** 10.1186/s12959-023-00580-6

**Published:** 2024-01-17

**Authors:** Mohammad A. Mohammad, Sophie Featherby, Camille Ettelaie

**Affiliations:** 1 Biomedical Sciences/Hull York Medial School, University of Hull, Cottingham Road, Hull, HU6 7RX UK; 2 Present address: The Department of Interdisciplinary Oncology, LSUHSC, New Orleans, LA 70112m USA

**Keywords:** Tissue factor, MAGI1, PAR2, PDZ domain, De-encryption, Thrombin generation activity, Proximity Ligation Assay, Co-immunoprecipitation

## Abstract

**Background:**

Tissue factor (TF) activity is stringently regulated through processes termed encryption. Post-translational modification of TF and its interactions with various protein and lipid moieties allows for a multi-step de-encryption of TF and procoagulant activation. Membrane-associated guanylate kinase-with inverted configuration (MAGI) proteins are known to regulate the localisation and activity of a number of proteins including cell-surface receptors.

**Methods:**

The interaction of TF with MAGI1 protein was examined as a means of regulating TF activity. MDA-MB-231 cell line was used which express TF and MAGI1, and respond well to protease activated receptor (PAR)2 activation. Proximity ligation assay (PLA), co-immunoprecipitation and pull-down experiments were used to examine the interaction of TF with MAGI1-3 proteins and to investigate the influence of PAR2 activation. Furthermore, by cloning and expressing the PDZ domains from MAGI1, the TF-binding domain was identified. The ability of the recombinant PDZ domains to act as competitors for MAGI1, allowing the induction of TF procoagulant and signalling activity was then examined.

**Results:**

PLA and fluorescence microscopic analysis indicated that TF predominantly associates with MAGI1 and less with MAGI2 and MAGI3 proteins. The interaction of TF with MAGI1 was also demonstrated by both co-immunoprecipitation of TF with MAGI1, and co-immunoprecipitation of MAGI1 with TF. Moreover, activation of PAR2 resulted in reduction in the association of these two proteins. Pull-down assays using TF-cytoplasmic domain peptides indicated that the phosphorylation of Ser253 within TF prevents its association with MAGI1. Additionally, the five HA-tagged PDZ domains of MAGI1 were overexpressed separately, and the putative TF-binding domain was identified as PDZ1 domain. Expression of this PDZ domain in cells significantly augmented the TF activity measured both as thrombin-generation and also TF-mediated proliferative signalling.

**Conclusions:**

Our data indicate a stabilising interaction between TF and the PDZ-1 domain of MAGI1 and demonstrate that the activation of PAR2 disrupts this interaction. The release of TF from MAGI1 appears to be an initial step in TF de-encryption, associated with increased TF-mediated procoagulant and signalling activities. This mechanism is also likely to lead to further interactions and modifications leading to further enhancement of procoagulant activity, or the release of TF.

**Supplementary Information:**

The online version contains supplementary material available at 10.1186/s12959-023-00580-6.

## Introduction

Tissue factor (TF) initiates the coagulation mechanism through formation of a complex with factor VIIa (fVIIa) which then activates factors X and IX [1, 2]. However, TF activity is stringently regulated to prevent inappropriate coagulation. The processes involved in the regulation of TF procoagulant activity include post-translational modification of TF as well as interaction with protein and lipid moieties on the surface of the cell. These mechanisms ensure that the de-encryption of TF activity  is strictly controlled [3–7]. Consequently, it has been suggested that under resting conditions, TF may exist in cryptic pools that render the protein unavailable to the environment surrounding the cells, in order to prevent inappropriate blood coagulation [8].

Membrane-associated guanylate kinase with inverted configuration (MAGI) are a group of proteins that are involved in scaffolding through multiprotein complex formation [9–12] and are also involved in the maintenance of the cell junctions [13–15]. To date, four MAGI proteins have been identified (MAGI1-3 and MAGIX). The MAGI proteins contain a number of separate protein binding motifs including WW and PDZ domains, with the latter being involved in binding of MAGI to c-terminus of various proteins [16]. MAGI proteins have been implicated in the control of cell migration and invasion and can modulate the activity of PTEN and Akt signalling, particularly during cancer [16–19].

In this study, the association of TF with MAGI proteins, before and after activation of PAR2, was examined in MDA-MB-231 cells. By identifying the TF-binding domain within MAGI1, the outcome of TF release from MAGI1, on the procoagulant activity was explored, and a novel mechanism for the regulation of TF availability is proposed.

## Material and methods

### Cell culture, cellular activation and determination of cell numbers

The MDA-MB-231 cell line (ATCC, Teddington, UK) was used due to the established and reliable patterns of TF and PAR2 expression and the response to PAR2 activation [20]. Initial analysis also indicated the presence of MAGI proteins in these cells [21]. The cells were cultured in DMEM (Lonza, Cambridge, UK) and supplemented with foetal calf serum 10% (v/v). Cells were seeded out as described and activated by incubation with PAR2-agonist peptide (PAR2-AP; SLIGKV; 20 µM) for durations stated in the results section. Cell numbers were determined by crystal violet staining as previously described [22] and calculated from a standard curve.

### Prediction of the PDZ binding domain within the cytoplasmic domain of TF and identification of potential binding proteins

The analysis of the possible PDZ binding domains within the cytoplasmic domain of TF was carried out using the POW web-site program and the scores for possible binding proteins determined [23].

### Duolink Proximity Ligation (PLA) assay

The procedure was carried out using the Duolink reagents (Sigma Chemical Co. Ltd, Poole, UK) and adapted from that previously described in detail [24] with minor modifications. MDA-MB-231 cells (10^3^) were seeded out into 35 mm-glass based μ-dishes (InVitro Scientific/Cellvis, Sunnyvale, USA) and adapted to serum-free medium for 1 h prior to activation. Sets of cells were then incubated with PAR2-AP (20 μM) for up to 40 min and then fixed with 4% (v/v) paraformaldehyde for 15 min. The cells were then washed three times with PBS and permeabilised with Triton X-100 0.1% (v/v) in PBS, for 5 min. All samples were blocked with Duolink blocking buffer for 1 h and incubated overnight with combinations of antibodies at 4 °C. In order to assess the proximity and potential interaction between TF and MAGI1-3, a mouse anti-TF antibody (HTF1; 5 μg/ml; eBioscience/Thermo Scientific, Warrington, UK) was used together with a rabbit anti-MAGI1 antibody (H-70; 2 μg/ml; Santa Cruz Biotechnology, Heidelberg, Germany), a rabbit anti-MAGI2 antibody (2 μg/ml; GenTex/Insight Biotechnologies, Wembley, UK) or a rabbit anti-MAGI3 antibody (2 μg/ml; Novus/R&D Systems, Abingdon, UK). In other experiments, the proximity between TF and the overexpressed PDZ-domains of MAGI1 were analysed using a rabbit anti-HA tag (C2954; Cell Signalling Technologies/New England Biolabs, Hitchin, UK) which was used at 1 µg/ml. The antibodies were diluted in the provided antibody diluent and the samples were blocked with the provided blocking buffer. A positive control was prepared using mouse anti-TF antibody (HTF1; 5 μg/ml) and a rabbit polyclonal anti-TF antibody (FL295; 5 μg/ml; Santa Cruz Biotechnology). Additionally, as negative controls, the antibodies were substituted with rabbit or mouse IgG isotypes (New England Biolabs; 2 μg/ml and 5 µg/ml respectively). The cells were washed three times with PBS and PLA performed according to the manufacturer’s instructions. The cells were then stained with DAPI (2 μg/ml) and Phalloidin-FITC (2 µg/ml). Images were acquired using a Zeiss Axio Vert.A1 inverted fluorescence microscope with a × 40 magnification (Carl Zeiss Ltd, Welwyn Garden City, UK). The number of red fluorescent events and nuclei were determined using ImageJ, in 10 fields of view from each assay [24].

### Co-immunoprecipitation of proteins, SDS-PAGE and western blot analysis

Cells were lysed in PhosphoSafe Extraction Reagent (Sigma) containing a protease inhibitor cocktail and cell debris removed by centrifugation. TF and MAGI1 proteins were immunoprecipitated from cell lysates (2 × 10^5^) using the anti-TF (HTF-1; 4 µg; eBioscience/Thermo Scientific, Warrington, UK) and anti-MAGI1 (H-70; 4 µg; Santa Cruz) antibodies respectively. To ensure specificity, relevant IgG isotypes (4 µg) were also included as well as additional controls without any antibody. All samples were incubated at 4 °C overnight with gentle shaking. Pureproteome protein A-magnetic beads (10 µl) (Merck-Millipore, Watford, UK) was added to all samples and controls and incubated at 4 °C for 90 min. The samples were then placed in a magnetic stand and the supernatant removed, washed five times with PBST (1 ml) and the samples denatured in SDS-PAGE loading buffer (70 µl) (Sigma). The samples were separated by 12% (w/v) SDS-PAGE, transferred onto nitrocellulose membranes and then blocked with TBST (10 mM Tris–HCl pH 7.4, 150 mM NaCl, 0.05% Tween-20). When immunoprecipitating with anti-TF, the membranes were probed using anti-MAGI1 (H-70) and a polyclonal rabbit anti-TF antibody (FL295; Santa Cruz) diluted 1:4000 (v/v) in TBST. When immunoprecipitating with anti-MAGI1, the membranes were probed with an anti-TF antibody (HTF1) and a mouse anti-MAGI1 antibody (SS-5; Santa Cruz). In some experiments, immunoprecipitation was carried out from transfected MDA-MB-231 cells using a rabbit anti-HA antibody (C2954; 4 µg; Cell Signalling Technologies/New England Biolabs) and the presence of TF examined using the anti-TF antibody (HTF1). The membranes were then washed three times with TBST and probed with a goat anti-mouse or goat-anti-rabbit alkaline phosphatase-conjugated antibody (Santa Cruz Biotechnology), diluted 1:4000 (v/v), and incubated for 90 min. Bands were then visualised using the Western Blue stabilised alkaline phosphatase-substrate (Promega Corp., Southampton, UK), recorded and analysed using ImageJ program.

### Pull-down assay using immobilised TF peptides

The plate pull-down procedure used was an adaptation of the method described and confirmed previously [25]. Substrate peptides, corresponding to the last 18 amino acids of the cytoplasmic domain of TF were synthesised in biotinylated form (Biomatik, Ontario, Canada). In total 4 peptides were synthesised in non-phosphorylated, single-phosphorylated (phospho-253 or phospho-258) and double-phosphorylated (phospho-253 and 258) forms and used as bait. A scrambled peptide (biotin-SWGNVSKLSAPRQGVNKE) was also included alongside as a negative control. TF peptides (5 µM final concentration) were diluted with PBS and distributed (100 µl per well) into a NeutrAvidin-coated 96-well plate (Thermo Scientific, Warrington, UK) and incubated for 2 h at room temperature to allow binding. The wells were then washed four times with PBST (300 µl). Cell lysates from non-activated and PAR2-activated cells (100 µl from 2 × 10^5^ cells) were incubated in the plates for 1 h at room temperature. The wells were then washed four times and probed with rabbit anti-MAGI1 (H-70; Santa Cruz) or mouse anti-MAGI3 (46; Santa Cruz) antibodies diluted 1:200 (v/v) in PBST. The samples were detected using goat anti-rabbit and goat anti-mouse horseradish peroxidase-conjugated antibodies (Santa Cruz), diluted 1:200 (v/v) and the colour developed with TMB One Solution (100 µl) (Promega). Once the colour was developed the reactions were stopped by the addition of 2 M sulphuric acid (50 µl) and absorptions measured at 450 nm using a plate reader.

### Cloning, transfection and expression of PDZ domains of MAGI1

The cloned cDNA for MAGI1 (pDONR223-MAGI1) was obtained from Addgene (Cambridge, UK) and the regions corresponding to PDZ1-5 were separately amplified using the forward and reverse primers as shown in Supplementary Table 1. Following digestion with BamHI and HindIII, the cDNA was sub-cloned into the FLAG-HA-pcDNA3.1 (Addgene) and positive colonies confirmed by sequencing (Eurofin-MWG, Wolverhampton, UK). In addition, two DNA regions encompassing the N-terminal of MAGI1, including or excluding PDZ1, were also cloned as above, using the primers shown in Supplementary Table 1. The sequences of the cloned DNA plasmid constructs were then verified as above. MDA-MB-231 cells (2 × 10^5^) were seeded out into 48-well plates and transfected with 0.5 µg of the plasmids to express the individual PDZ domains, or the two longer N-terminal-spanning regions. Transfection of the cells was carried out using TransIT-2020 (Geneflow, Lichfield, UK) according to the manufacturer's instructions. Cells were permitted to express the proteins for 48 h and the expression of the proteins were confirmed by western blot using rabbit anti-HA antibody. The associations of the overexpressed MAGI1-PDZ domains, or the MAGI-N-terminal domains with the endogenous TF were then analysed in situ, by PLA. An attempt to sub-clone the entire MAGI1 was unsuccessful due to the large size of the insert DNA.

Additionally, in an attempt to suppress the expression of MAGI1, the cells were transfected with a specific siRNA for MAGI1 (100 nM; SilencerSelect siRNA, ThermoFisher UK, Loughborough, UK) using TransIT-2020 reagent, according to the manufacturer’s instructions. The cells were incubated up to 48 h but were not viable for experimentation.

Finally, in some experiments, cells were transfected with a pCMV-AC-TF-tGFP plasmid (OriGene, Rockville, USA) which encodes for turbo-GFP (tGFP) in tandem, on the C-terminal of TF which was mutated to substitute aspartate instead of serine-253 [26]. Cells (2 × 10^5^) were transfected with 1 μg of plasmid DNA as above and permitted to express the protein over 48 h. The cells were then lysed and the TF-tGFP immunoprecipitated with anti-tGFP-magnetic beads (clone 2H8) (25 μl) according to the manufacturer's protocol (OriGene) at 4 °C. The samples were then examined as above for the presence of MAGI1.

### Cell-based thrombin-generation assay

Cell surface TF-fVIIa activity was measured by modification of a previously described procedure [25]. In some experiments, to confirm that the activity was attributed to TF, the cells were pre-incubated with an inhibitory anti-TF antibody (HTF1; 20 µg/ml) before analysis. All cells (10^5^) were washed with the reaction buffer (Tris-buffered saline (TBS) pH 7.4), containing 1% (w/v) bovine serum albumin (BSA) and then incubated for 20 min at 37 °C with a mixture of barium sulphate absorbable proteins (2 mg/ml; Sigma Chemical Company Ltd, Poole, UK) and 5 mM CaCl_2_ in the reaction buffer (total volume 150 µl). Aliquots of reaction (100 µl) were then transferred to 96-well plates containing 100 µl of thrombin substrate CS-01(38) (0.2 µM H–D-Phe-Pip-Arg-pNA; Hyphen BioMed/Quadratech, Epsom, UK) and incubated for a further 40 min at 37 °C. The reactions were stopped by the addition of 2% (v/v) acetic acid (50 µl) and the absorption of the sample measured at 410 nm on a plate reader. TF activity was determined from a standard curve prepared alongside.

### Fluorescence microscopic examination of cellular TF and MAGI1

MDA-MB-231 cells were analysed by fluorescence microscopy, before and after activation with PAR2-AP. The cells were fixed and probed for TF using an FITC-conjugated anti-TF antibody (HTF1-FITC; Miltenyi Biotec, Woking, UK). The cells were also probed with a rabbit anti-MAGI1 antibody (H-70) and then labelled with a goat anti-rabbit IgG-AlexaFluor 594 antibody (Life Technologies, Paisley, UK). The samples were examined by fluorescence microscopy and the co-localisation overlap coefficient values determined.

### Measurement of ERK1/2 phosphorylation, Akt phosphorylation, Cyclin D mRNA expression and cell proliferation

In order to determine the outcome of expression of the MAGI1 peptides, MDA-MB-231 cells were transfected to express the N-terminal regions with, or without the PDZ1 domain and the influence on various indicators of cell proliferation and survival were examined. In the first experiment, the cells were lysed and the phosphorylation of Akt was compared in the two transfected samples. The cells (10^5^) were lysed in Laemmli buffer (Sigma) and separated on denaturing 12% (w/v) polyacrylamide gels. Proteins were transferred onto nitrocellulose membranes and blocked with TBST (10 mM Tris–HCl pH 7.4, 150 mM NaCl, 0.05% Tween-20) for 1 h. The membranes were probed with a goat anti-human Akt1/2 (N-19) polyclonal antibody and a rabbit polyclonal anti-human Akt1 (phospho-S473). The membranes were then washed three times with TBST and probed with a donkey anti-goat, or goat anti-rabbit alkaline phosphatase-conjugated antibody (Santa Cruz), diluted 1:4000 (v/v), and incubated for 90 min. Bands were then visualised using the Western Blue stabilised alkaline phosphatase-substrate (Promega), recorded and analysed using ImageJ program.

Next, cells (10^5^) were transfected as above and one set was pre-incubated with a monoclonal antibody (SAM11; 20 µg/ml; Santa Cruz Biotechnology, Heidelberg, Germany) throughout culturing, in order to block PAR2 activation by TF activity. The cells were then lysed and proteins separated by SDS-PAGE as above. Western blot analysis of ERK1/2 phosphorylation in the samples was carried out using an anti-phosphoT202/185-phosphoY204/187-ERK1/2 antibody (Cell Signalling Technology/New England Biolabs, Hitching, UK) and total ERK1/2 was detected using an anti-ERK1/2 antibody (Cell Signalling/NEB) diluted 1:3000 (v/v) in TBST. GAPDH was also detected using a rabbit anti-GAPDH polyclonal antibody (V-18; Santa Cruz Biotechnology, Heidelberg, Germany) diluted 1:5000 (v/v) in TBST. The membranes were then probed with a goat anti-rabbit, or donkey anti-goat alkaline phosphatase-conjugated antibody (Santa Cruz), diluted 1:5000 (v/v) in TBST. Bands were then visualised using the Western Blue stabilised alkaline phosphatase-substrate, recorded and analysed using ImageJ program.

To further demonstrate the influence and the effectiveness of inclusion of PDZ1 within the N-terminal of MAGI1, cells were transfected as above and pre-incubated with a monoclonal antibody (SAM11; 20 µg/ml) capable of blocking PAR2 activation, or a mouse monoclonal antibody to inhibit the protease activity of TF-fVIIa complex (HTF1; 20 µg/ml; eBioscience/Thermo Scientific, Warrington, UK) [27] or a mouse IgG isotypes (New England Biolabs). Total RNA was isolated using the Monarch total RNA extraction kit (New England Biolabs, Hitchin, UK) from 10^5^ cells. Samples (100 ng) were amplified using the primers 5’- CCG TCC ATG CGG AAG ATC -3’ (forward) and 5’- ATG GCC AGC GGG AAG AC -3’ (reverse) [28]. The reaction was carried out at an annealing temperature of 60 °C for 1 min using the GoTaq® 1-Step RT-qPCR System (Promega Corporation Ltd, Southampton, UK) on an iCycler thermal cycler (Bio-Rad, Hemel Hempstead, UK) for 40 cycles. Following amplification, the relative amounts of target mRNA were determined using the 2^−ΔΔCT^ method [29]. Finally, cell numbers were determined using the crystal violet measurement as described above.

### Statistical analysis

Presented data include the calculated mean values ± the calculated standard error of the mean. Statistical analysis was carried out using the GraphPad Prism version 9.0 (GraphPad Software, Boston, Massachusetts USA). Significance was determined using one-way ANOVA (analysis of variance) and Tukey’s honesty significance test or where appropriate, by paired t-test and *p* values of equal or less than 0.05 were deemed to be significant.

## Results

### TF associates with MAGI1 protein and the interaction is reduced following PAR2 activation

**Fig. 1:**
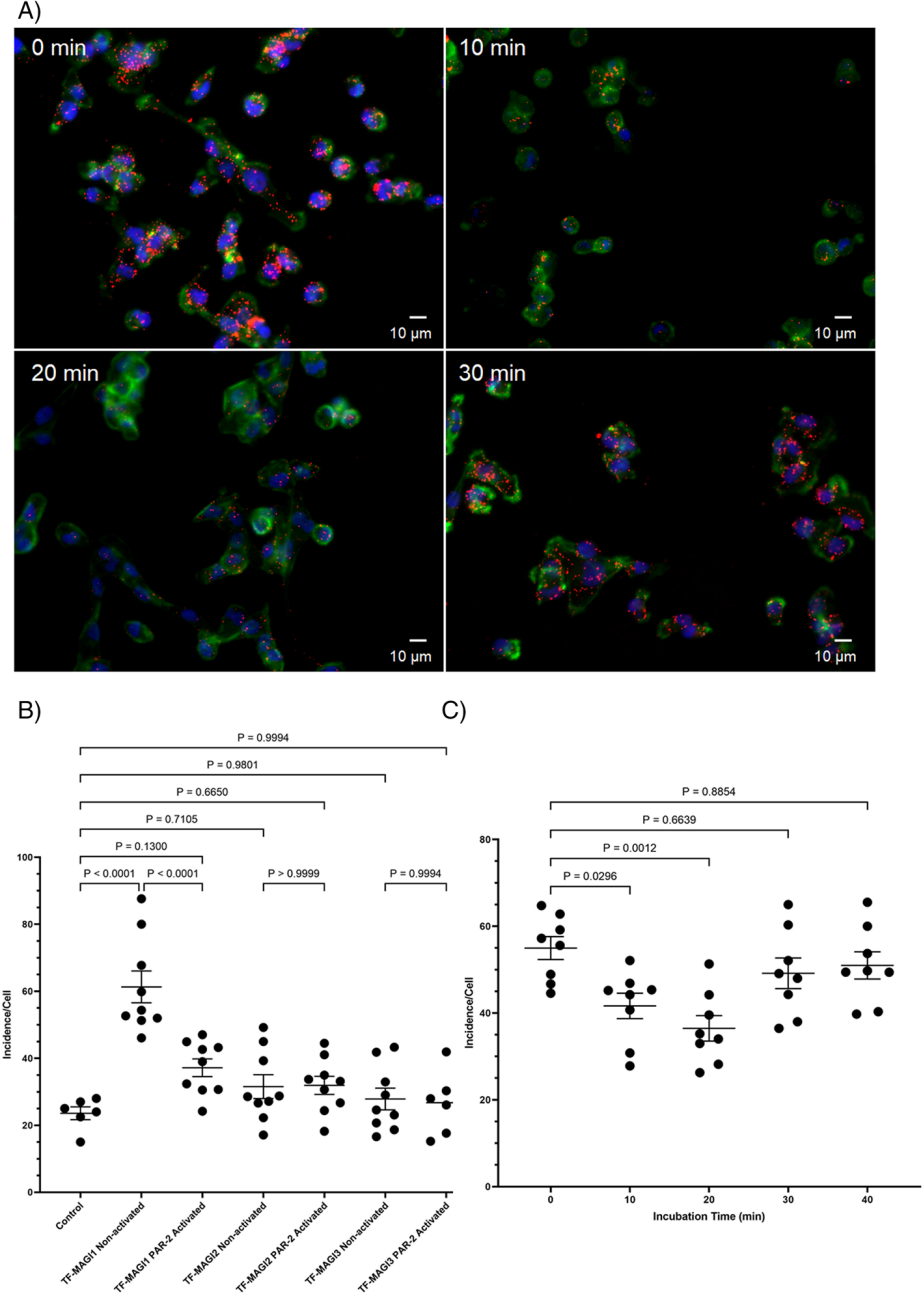
Analysis of the interaction of TF and MAGI1-3 by proximity ligation assay and the influence of PAR2 activation. MDA-MB-231 cells (10^3^) were seeded out into 35 mm-glass based μ-dishes and adapted to serum-free medium for 1 h prior to activation. The cells were then incubated with PAR2-AP (20 μM) for up to 30 min and then fixed with 4% (v/v) paraformaldehyde for 15 min. The cells were washed three times with PBS and permeabilised with Triton X-100 0.1% (v/v) in PBS, for 5 min. All samples were blocked with Duolink blocking buffer for 1 h and incubated overnight with combinations of antibodies as follows, at 4 °C. The proximity between TF and MAGI1-3 were examined using a mouse anti-TF antibody HTF1 (5 μg/ml) together with a rabbit anti-MAGI1 antibody (H-70; 2 μg/ml), a rabbit anti-MAGI2 antibody (2 μg/ml) or a rabbit anti-MAGI3 antibody (2 μg/ml). The antibodies were diluted in the provided antibody diluent and blocked with the provided blocking buffer. The cells were washed three times with PBS and PLA performed according to the manufacturer’s instructions. The cells were labelled with DAPI (2 μg/ml) and Phalloidin-FITC (2 µg/ml). Images were acquired using a Zeiss Axio Vert.A1 inverted fluorescence microscope with a × 40 magnification. (The micrographs are representative of 5 fields of view from 9 experiments, RED = PLA incidences; GREEN = Phalloidin; BLUE = DAPI). **B** The interactions of TF with MAGI1-3 in non-activated and at 20 min post-activation was analysed. **C** The interaction of TF and MAGI1 at intervals up to 40 min was analysed by PLA

The prediction of possible PDZ binding domains in the cytoplasmic domain of TF, carried out using the POW web-site program, produced high scores for PDZ domains in MAGI1, MAGI3, MAGIX proteins and also Rhophilin-1 and also identified the sequence ENSPL within the c-terminus of TF as a possible target (Supplementary Table 2). Examination of the association of TF with MAGI1-3 was carried out by PLA. The level of reactions were compared to a positive control employing two antibodies against TF and also a negative control in which an IgG isotype was used for one of the antibodies (Supplementary Fig. 1). Examination of the data showed the greatest level of association with MAGI1 (Fig. 1A and B). The activation of cells using PAR2-AP resulted in 26% reduction in TF-MAGI1 proximity but the change was not significant for MAGI2 and MAGI3 (Fig. 1B). To examine the time-course of TF-MAGI1 dissociation, PLA analysis was carried out on PAR2-activated MDA-MB-231 cells over 40 min. Analysis of the cells indicated a transient decrease in the TF-MAGI1 association at around 20 min which then partially normalised by 30 min post-activation (Fig. 1A and C).

**Fig. 2:**
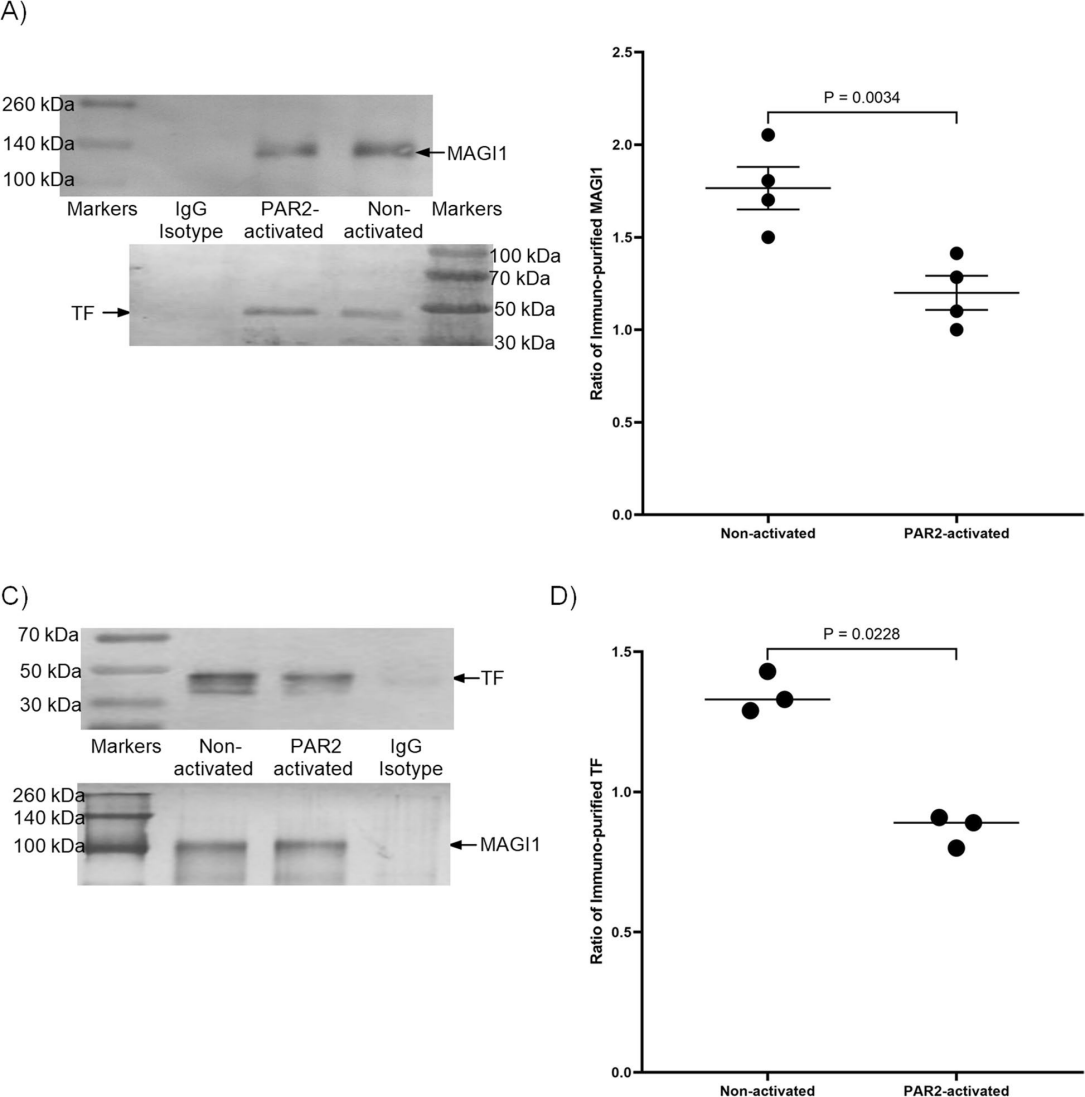
Analysis of the interaction of TF and MAGI1-3 and the influence of PAR2 activation. **A** MDA-MB-231 cells (2 × 10^5^) were adapted to serum-free medium for 1 h prior to activation. The cells were then incubated with PAR2-AP (20 μM) for up to 20 min and TF was immunoprecipitated from cell lysates with the anti-TF (HTF-1; 4 µg) antibody using protein A-magnetic beads. The samples were washed five times with PBST (1 ml) and denatured in SDS-PAGE loading buffer and examined for MAGI1 and TF by western blot using an anti-MAGI1 (H-70) and a rabbit anti-TF antibody (FL-295). **B** The ratio of the MAGI1 band densities were normalised against those of TF in the same co-immunoprecipitated samples. **C** In addition, MAGI1 was immunoprecipitated from cell lysates with an anti-MAGI1 (H-70; 4 µg) antibody using protein A-magnetic beads. The samples were washed five times with PBST (1 ml) and denatured in SDS-PAGE loading buffer and examined for TF and MAGI1 by western blot using an anti-TF antibody (HTF-1) and a mouse anti-MAGI1 antibody (SS-5). **D** The ratios of the TF band densities were normalised against those of MAGI1 in the same co-immunoprecipitated samples

To confirm the interaction between TF and MAGI1, an attempt was made to co-immuno-purify MAGI1 with TF. Western blot analysis of anti-TF immuno-purified proteins, probed with a rabbit anti-MAGI1 antibody, identified a high molecular weight band (Fig. 2A and B). Moreover, the quantification of the amount of detected MAGI1 agreed with the PLA data showing a reduction in the binding of TF and MAGI1 (33%) following PAR2 activation of the cells. Consequently, immunoprecipitation was carried out in reverse using anti-MAGI1 antibody, and the presence of TF explored by western blot. Analysis of these samples indicated a significant level of association between MAGI1 and TF and also agreed with the above data, indicating a 19% drop in the amount of co-purified TF following PAR2 activation of the cells (Fig. 2C and D). These data, confirm those obtained by PLA and clearly show an association between TF and MAGI1 which may be disrupted following the activation of PAR2. Co-immunoprecipitation studies using anti-MAGI2, anti-MAGI3, anti-MAGIX (D-18) and anti-Rhophilin-1 (I-19) did not indicate detectable protein interactions (Supplementary Fig. 2). The fractions prepared by immunoprecipitation of TF_Asp253_-tGFP-using anti-tGFP-magnetic beads did not indicate the presence of MAGI1 when examined by western blots (not shown). However, no conclusions regarding the effect of phosphorylation on TF-MAGI1 interaction were derived from this experiment.

**Fig. 3:**
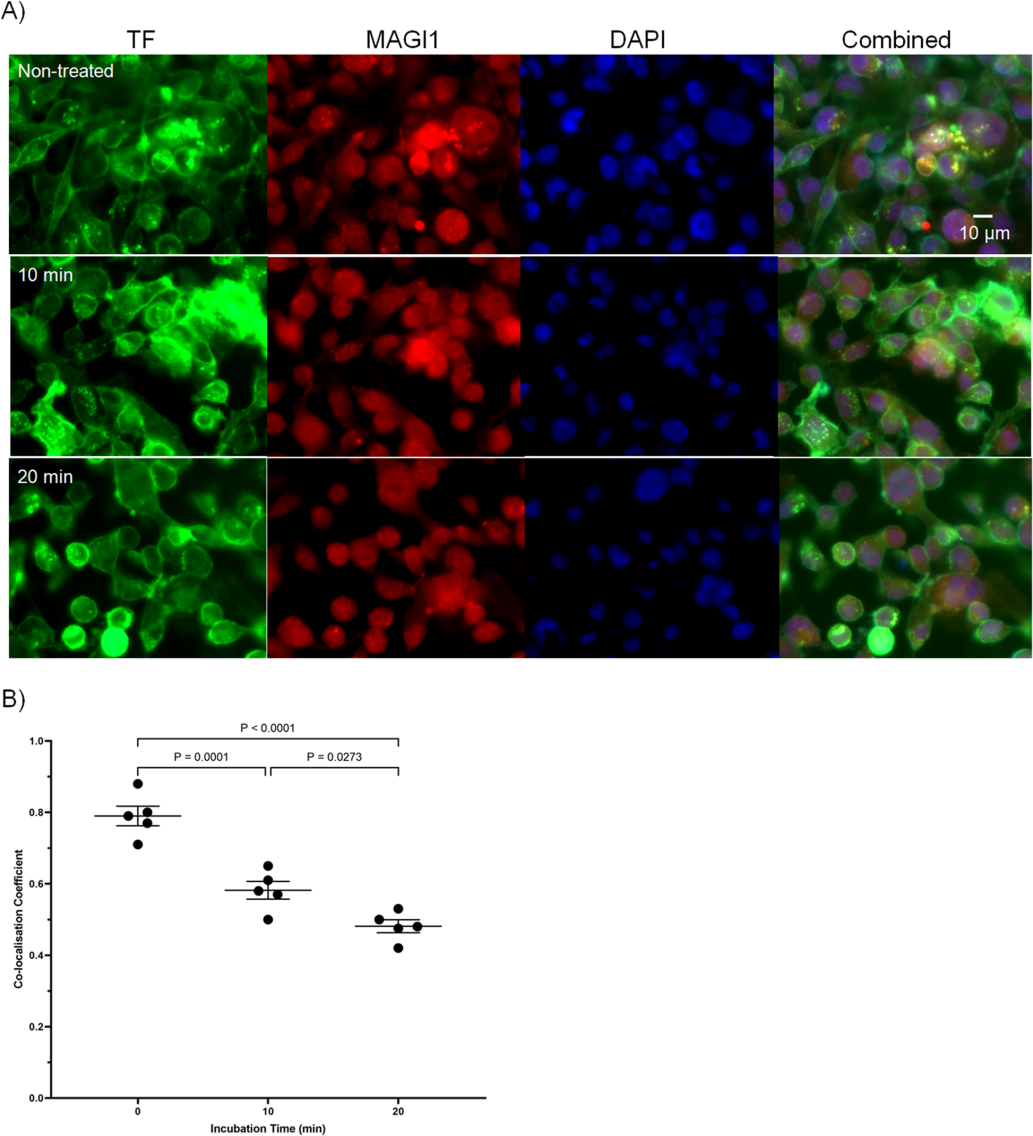
Qualitative co-localisation of TF and MAGI1 in resting and activated MDA-MB-231 cells. MDA-MB-231 cells (10^4^) were seeded out into 35 mm-glass based μ-dishes and sets were activated by incubation with PAR2-AP (20 μM). The cells were fixed and probed for TF using an FITC-conjugated anti-TF antibody (HTF1-FITC). The patterns were compared to MAGI1 probed with a rabbit anti-MAGI1 antibody (H-70) and developed a goat anti-rabbit IgG-AlexaFluor 594 antibody. The cells were also stained with DAPI (2 μg/ml) and examined by fluorescence microscopy. Co-localisation coefficient values were then determiend using the ImageJ program. The values were calculated as the average from 10 captured images, and the data show the values for 5 separate experiments

The co-localisation of TF with MAGI1, and the disruption following activation with PAR2 was also examined qualitatively, by fluorescence microscopy. Pre- and post-activated MDA-MB-231 cells were fixed and probed for TF using a FITC-conjugated anti-TF antibody (HTF1-FITC) and anti-MAGI1 antibody (H-70). Analysis of the labelled cells suggests an association (overlap coefficient = 0.791) with MAGI1 (Fig. 3). Furthermore, activation of PAR2 on cells resulted in a progressive reduction in TF-MAGI1 co-localisation (overlap coefficient = 0.48) at 20 min.

### Phosphorylation of Ser253 on TF prevents the interaction of TF with MAGI1

**Fig. 4:**
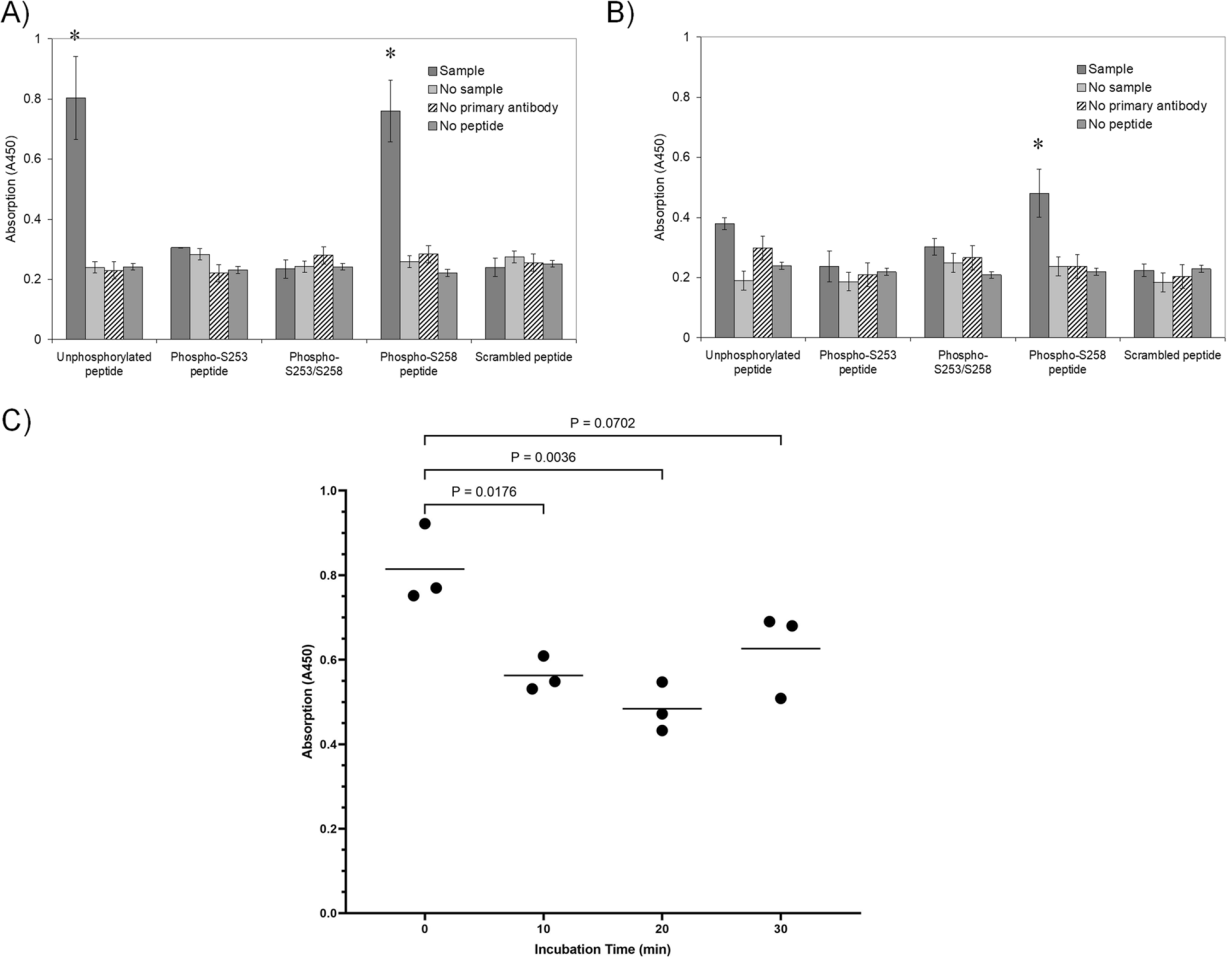
Examination of influence of TF phosphorylation on the binding of MAGI1 and 3. Biotinylated peptides, corresponding to the last 18 amino acids of the cytoplasmic domain of TF were synthesised in non-phosphorylated, single-phosphorylated and double-phosphorylated forms and used as bait. An additional scrambled peptide (biotin-SWGNVSKLSAPRQGVNKE) was also included alongside. The peptides (5 µM final concentration) in PBS, were bound into a NeutrAvidin-coated 96-well plate and then blocked. Cell lysates from resting and PAR2-activated MDA-MB-231 cells (from 2 × 10^5^ cells) were incubated in the plates for 1 h at room temperature. The wells were then washed four times and probed with (**A**) rabbit anti-MAGI1 (H-70) or (**B**) mouse anti-MAGI3 (46) antibodies diluted 1:200 (v/v) in PBST. The samples were detected using goat anti-rabbit and goat anti-mouse alkaline phosphatase-conjugated antibodies diluted 1:200 (v/v) and the colour developed with TMB One Solution (100 µl). Once the colour was developed the reactions were stopped and absorptions recorded. (*n* = 3; * = *p* < 0.05 vs. the respective samples without cell lysate). **C** The interaction of MAGI1 with the non-phosphorylated TF peptide was examined by pull-down over a period of 30 min following activation of PAR2, as described above

Previously, we showed that the activation of PAR2 on cells leads to the phosphorylation of Ser253 within the cytoplasmic domain of TF which was determined to peak at around 20 min post-activation in MDA-MB-231 cells [24]. Therefore, to examine the possible role of TF phosphorylation in the association between TF and MAGI1, pull-down assays were carried out using peptides corresponding to the cytoplasmic domain of TF with different phosphorylation states [24, 25]. 96-well plates were pre-coated with peptides corresponding to the cytoplasmic domain of TF in different phosphorylation states. The plates were incubated with the lysate from non-activated MDA-MB-231 cells, washed and probed with an anti-human MAGI1 or anti-human MAGI3 antibody. MAGI1 was shown to associate with the non-phosphorylated and the Ser258-phosphorylated TF peptides but not the Ser253-phosphorylated, double-phosphorylated or the scrambled peptides (Fig. 4A). MAGI3 associated with Ser258-phosphorylated peptide only, but not with the other TF peptides (Fig. 4B). A time-course analysis was carried out using MDA-MB-231 cells which were activated using PAR2-AP and incubated for up to 30 min. Pull-down of MAGI1 using the non-phosphorylated TF cytoplasmic peptide, from the lysates corresponding to different time-points showed a reduction in the level of purified MAGI1 (39%) at around 20 min which agrees with the above data (Fig. 4C).

### Characterisation of the interaction of TF and MAGI1

**Fig. 5:**
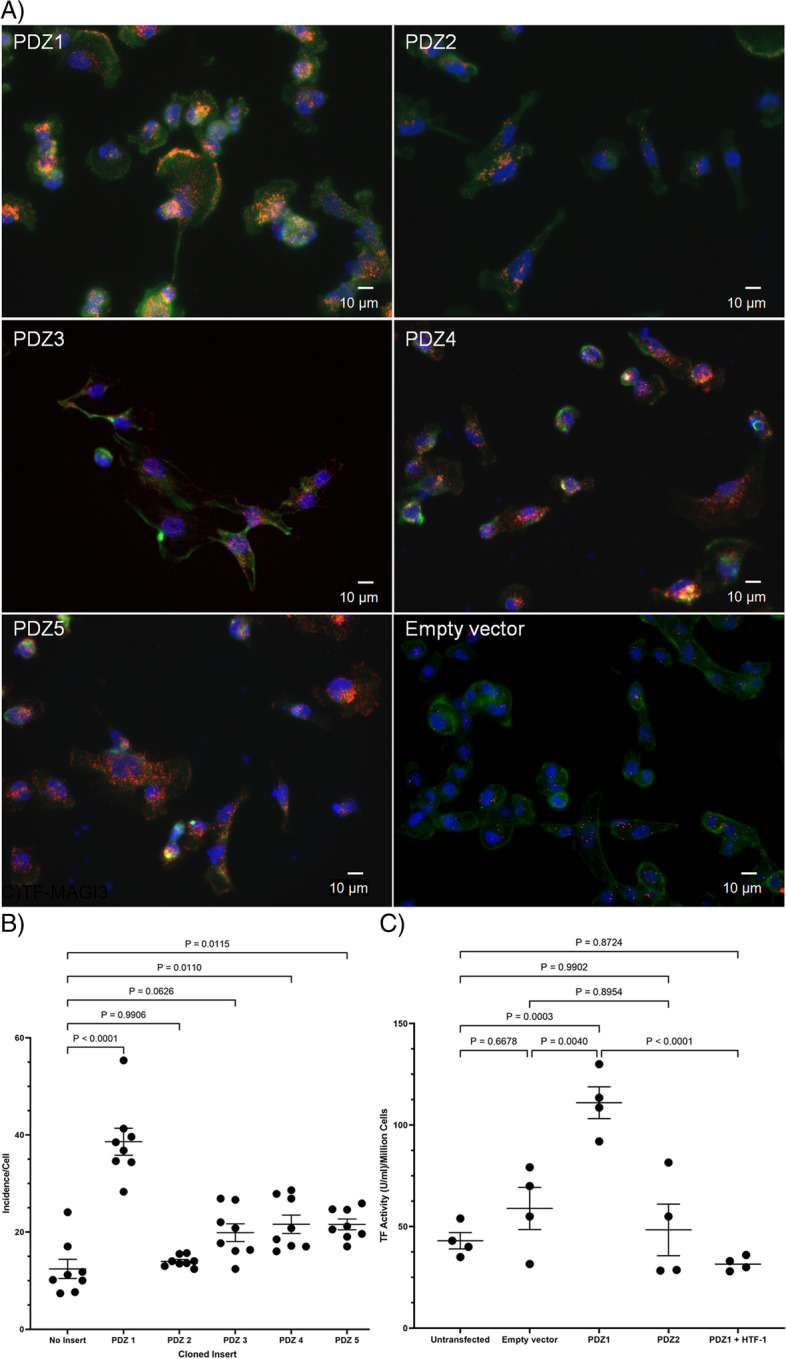
Identification of the PDZ domain within MAGI1 responsible for binding TF. MDA-MB-231 cells (10^3^) were seeded out into 35 mm-glass based μ-dishes and separately transfected with constructs to express PDZ1-5 of from MAGI1. The cells permitted to express the proteins for 24 h and then fixed and washed. **A** The proximity of the expressed PDZ to TF was examined by PLA using a rabbit anti-HA (C2954) antibody and a mouse anti-TF (HTF1) antibody, respectively (RED = PLA incidences; GREEN = Phalloidin; BLUE = DAPI) and (**B**) quantified using the ImageJ program. **C** Cell surface TF-fVIIa activity was measured on the transfected cells expressing PDZ1, PDZ2 or the empty vector, using a modified thrombin-generation assay. An additional sample of cells were transfected to express PDZ1 but were pre-incubated with an inhibitory anti-TF antibody (HTF1; 20 µg/ml) before analysis. The cells (10^5^) were incubated for 20 min at 37 °C with a mixture of barium sulphate absorbable proteins (2 mg/ml) and 5 mM CaCl_2_ in the reaction buffer (total volume 150 µl; Tris-buffered saline pH 7.4), containing 1% (w/v) bovine serum albumin (BSA). Aliquots of reaction (100 µl) were then transferred to 96-well plates containing 100 µl of thrombin substrate CS-01(38) (0.2 µM H–D-Phe-Pip-Arg-pNA) and incubated for a further 40 min at 37 °C. The reactions were stopped by the addition of 2% (v/v) acetic acid (50 µl) and the absorption of the sample measured at 410 nm on a plate reader

An attempt to suppress the expression of MAGI1 by siRNA transfection of the cells resulted in low viability and detachment of the cells. Consequently, this approach was abandoned. Therefore, to further characterise the interaction between TF and MAGI1, HA-FLAG-tagged forms of PDZ1-5, derived from the cDNA of MAGI1 were individually assessed for their ability to associate with TF by PLA. Analysis was carried out using a mouse anti-TF antibody together with a rabbit anti-HA antibody (Fig. 5A). Analysis of the interaction of TF with MAGI1 PDZ domains showed the greatest association with PDZ1, and least with PDZ2. However, lower levels of association between TF and PDZ3, PDZ4 and PDZ5 were detectable (Fig. 5B). To analyse the influence of interaction of MAGI1 with TF on the activity of the latter protein, an attempt was made to compete out MAGI1 by the expression of PDZ1, and compare this to the influence of expression of the non-binding PDZ2. Examination of the thrombin-generation indicated a significantly higher TF activity on cells overexpressing PDZ1 (Fig. 5C) but was suppressed on pre-incubation with the inhibitory anti-TF (HTF1) antibody.

**Fig. 6:**
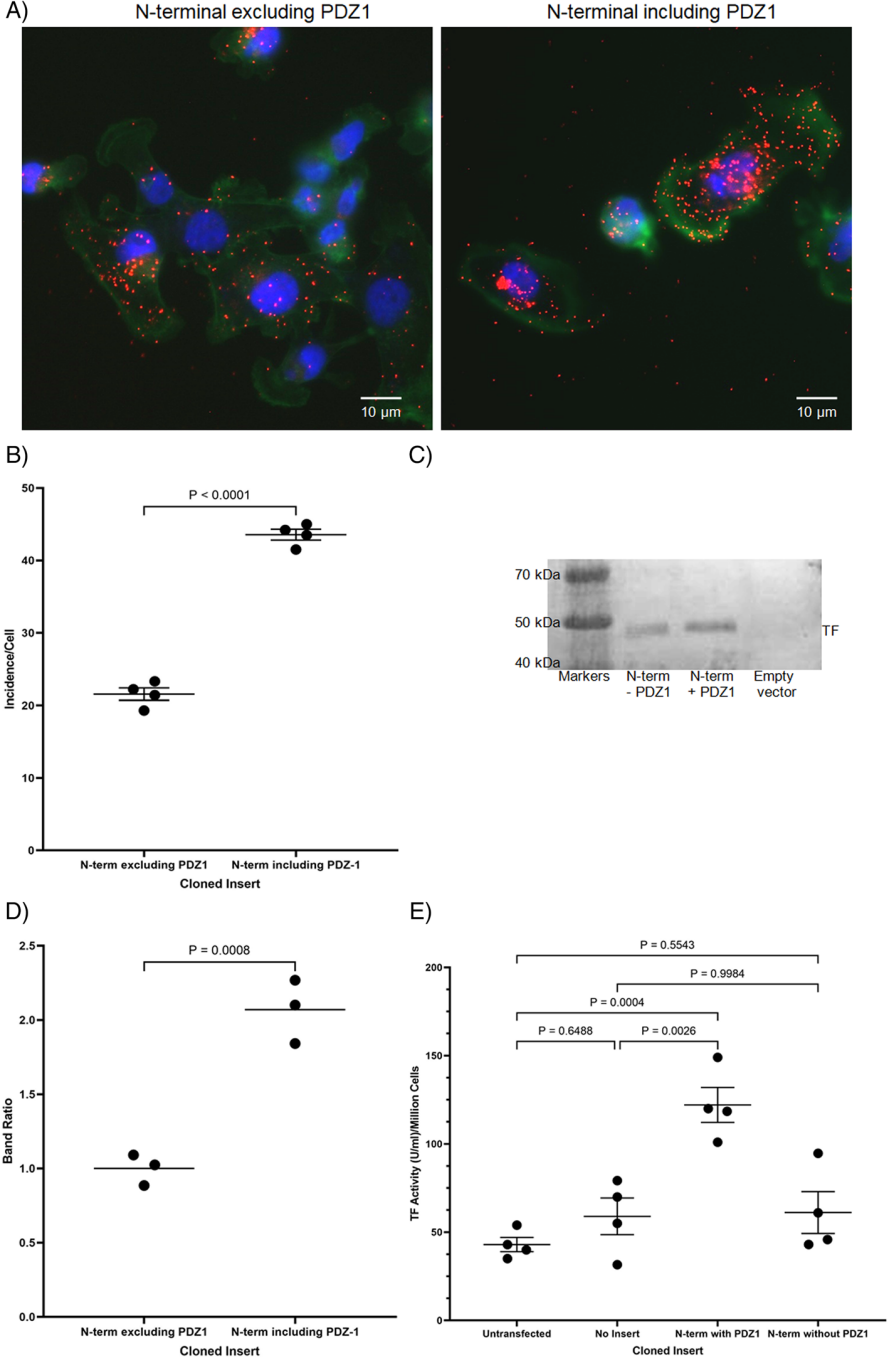
Examination of the role of PDZ1 in interaction with TF. MDA-MB-231 cells (10^3^) were seeded out into 35 mm-glass based μ-dishes and separately transfected with constructs to express the N-terminal domain of MAGI1 including and excluding PDZ1. The cells were permitted to express the proteins for 24 h and then fixed and washed. **A** The proximity of the expressed N-terminal peptides to TF was examined by PLA using a rabbit anti-HA (C2954) antibody and a mouse anti-TF (HTF1) antibody, respectively (RED = PLA incidences; GREEN = Phalloidin; BLUE = DAPI) and (**B**) quantified using the ImageJ program. **C** TF was immunoprecipitated from the lysates of the cells expressing the two N-terminal peptides (including and excluding PDZ1) using a mouse anti-HA antibody (C2954). The samples were then examined by western blot and probed using rabbit anti-TF antibody (HTF1). **D** The relative amounts of TF were then analysed in the samples. **E** Cell surface TF-fVIIa activity was measured on the transfected cells expressing the N-terminal of MAGI1 with and without the PDZ1 region, or the empty vector, using a modified thrombin-generation assay. The cells (10^5^) were incubated for 20 min at 37 °C with a mixture of barium sulphate absorbable proteins (2 mg/ml) and 5 mM CaCl_2_ in the reaction buffer (total volume 150 µl; Tris-buffered saline (TBS) pH 7.4), containing 1% (w/v) bovine serum albumin (BSA). Aliquots of reaction (100 µl) were then transferred to 96-well plates containing 100 µl of thrombin substrate CS-01(38) (0.2 µM H–D-Phe-Pip-Arg-pNA) and incubated for a further 40 min at 37 °C. The reactions were stopped by the addition of 2% (v/v) acetic acid (50 µl) and the absorption of the sample measured at 410 nm

In addition, the N-terminal domain of MAGI1, up to the start of PDZ1 (excluding PDZ1), or the end of PDZ1 (including PDZ1) were expressed in MDA-MB-231 cells. The proximity of the expressed N-terminal-MAGI1 peptides and TF were then examined using PLA (Fig. 6A). Examination of cells indicated a significantly higher association on inclusion of PDZ1 (Fig. 6B). To further confirm this, the cell extracts were immuno-precipitated using the anti-HA antibody (C2954) and any TF co-purified with the HA-N-terminal-MAGI1 peptide was examined by western blot, using the anti-TF (HTF1) antibody (Fig. 6C). Examination of these blots demonstrated more than twofold increase in the co-purification of TF on inclusion of PDZ-1, compared to MAGI1 peptide devoid of PDZ1 domain (Fig. 6D). Furthermore, overexpression of the N-terminal peptide augmented the level of thrombin-generation only when the PDZ1 domain was included (Fig. 6E).

### Influence of MAGI1 N-terminal peptides on proliferative signalling

**Fig. 7:**
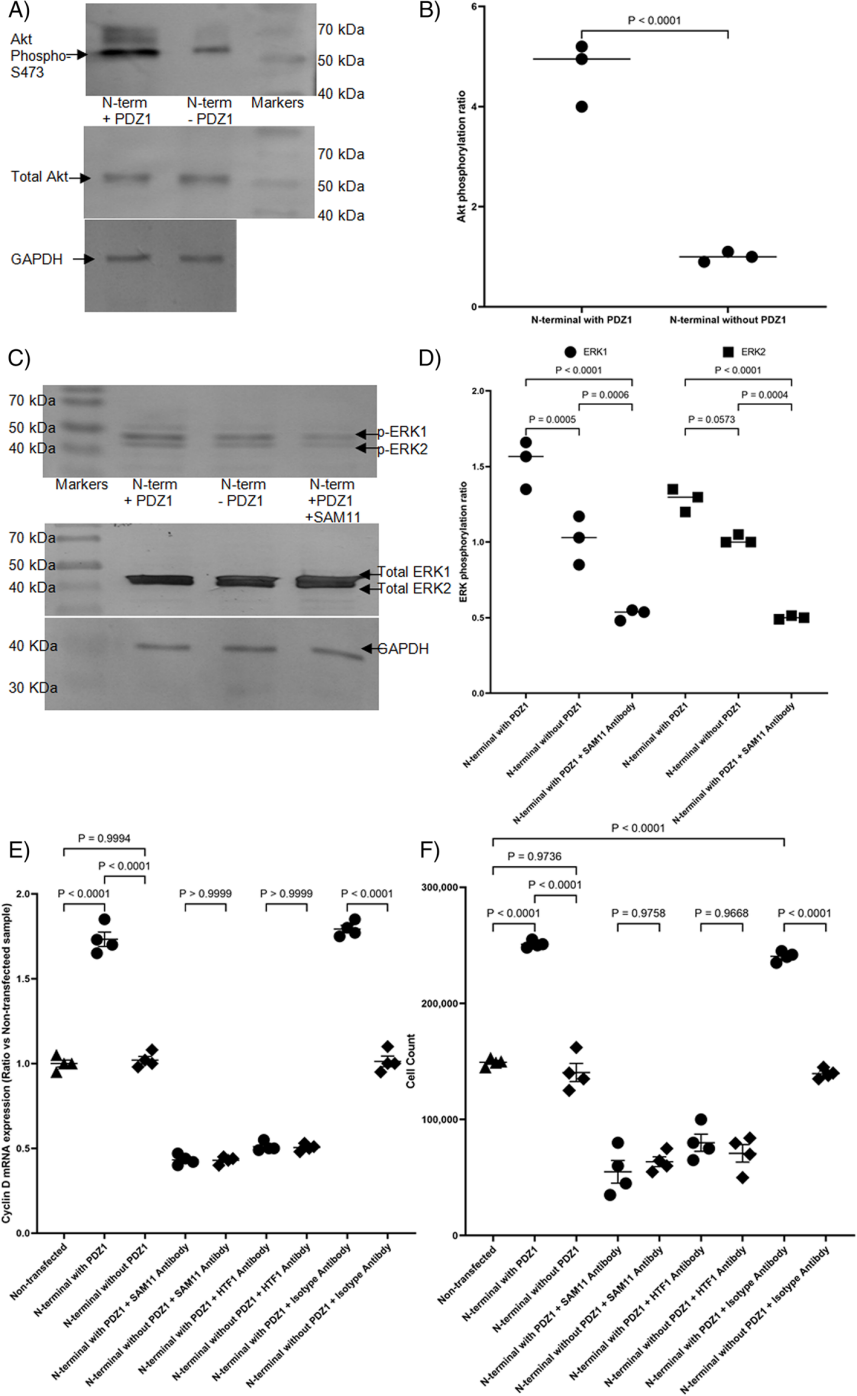
Outcome of expression of the MAGI1 N-terminal peptides on cell proliferation signalling. MDA-MB-231 cells (10^5^) were transfected to express the N-terminal of MAGI1 with and without the PDZ1 domain. Selected sets of cells were also pre-incubated with SAM11 antibody (20 µg/ml) as shown in the figures. The cells were lysed and proteins separated by denaturing 12% (w/v) polyacrylamide electrophoresis, transferred onto nitrocellulose membranes and blocked with TBST. **A** The membranes were probed with a goat anti-human Akt1/2 (N-19) polyclonal antibody and a rabbit polyclonal anti-human Akt1 (phospho-S473). The membranes were then washed and probed with a donkey anti-goat, or goat anti-rabbit alkaline phosphatase-conjugated antibody, diluted 1:4000 (v/v). Bands were visualised using the Western Blue stabilised alkaline phosphatase-substrate, recorded using ImageJ program and (**B**) the ratios calculated. **C** Separate sets of the western blot membrane were probed using an anti-phosphoT202/185-phosphoY204/187-ERK1/2 antibody or alternatively, total ERK1/2 was detected using an anti-ERK1/2 antibody diluted 1:3000 (v/v) in TBST. The membranes were also probed using a rabbit anti-GAPDH polyclonal antibody (V-18) diluted 1:5000 (v/v) in TBST. The membranes were then incubated with a goat anti-rabbit alkaline or a donkey anti-goat phosphatase-conjugated antibody diluted 1:5000 (v/v) in TBST and visualised as above, recorded using ImageJ program and (**D**) the ratios calculated. MDA-MB-231 cells (10^5^) were transfected to express the N-terminal of MAGI1 with and without the PDZ1 domain. Sets of cells were pre-incubated with a monoclonal antibody (SAM11; 20 µg/ml) to block PAR2 activation, or a mouse monoclonal antibody capable of inhibiting the protease activity of TF-fVIIa complex (HTF1; 20 µg/ml). **E** Total RNA was isolated from one set of the cells, and samples (100 ng) were amplified using the primers 5’- CCG TCC ATG CGG AAG ATC -3’ (forward) and 5’- ATG GCC AGC GGG AAG AC -3’ (reverse). The reaction was carried out at an annealing temperature of 60 °C using the GoTaq® 1-Step RT-qPCR for 40 cycles. Following amplification, the relative amounts of target mRNA were determined using the 2^−ΔΔCT^ method. **F** Cell numbers were determined in the second sets of cells using the crystal violet method

Since the expression of the N-terminal domain of MAGI1, including PDZ1, but not the peptide excluding PDZ1 augmented the TF activity, an attempt was made to examine the proliferative and pro-survival signals arising from the release of TF by the expression of the N-terminal peptide. Initially, cells were transfected to express the two peptides up to the start of PDZ1 (excluding PDZ1), or the end of PDZ1 (including PDZ1) in MDA-MB-231 cells. First, a substantial increase in the phosphorylation of Akt indicated a robust pro-survival signal in the on expression of the peptide which included PDZ1 (Fig. 7A and B). Furthermore, examination of ERK1/2 phosphorylation, as a marker for proliferative signalling indicated increased ERK1/2 phosphorylation on expression of the peptide including PDZ1, but this activity was significantly reduced on inhibition of PAR2 activation using the SAM11 monoclonal antibody (Fig. 7C and D). These observations were then further confirmed by more extensive measurement of the expression of cyclin D mRNA, as a more specific indicator of proliferative signalling and entry into cell cycle (Fig. 7E), which was also reflected in significant increases in cell numbers (Fig. 7F). Furthermore, pre-incubation of the cells with either SAM11 antibody to block PAR2 activation, or HTF1 antibody to prevent TF-fVIIa protease activity, suppressed both cyclin D expression and cell proliferation. Therefore collectively, these data indicate that the expression of MAGI1 N-terminal peptide with PDZ1 induced proliferative signalling through a mechanism involving the activation of PAR2, and requiring the protease activity of TF-fVIIa.

## Discussion

TF encryption is the post-translational suppression of TF procoagulant activity on the surface of cells. Unperturbed or non-activated cells present little TF activity despite the presence of TF antigen on the cell surface. The mechanism of TF de-encryption is not currently understood although at least four mechanisms have been suggested [3–7]. However, none of these alone is sufficient to explain the observed tight regulation of TF activity. Post-translational modification of TF alters the properties of TF resulting in alteration in the procoagulant and signalling activities as well as its release as microvesicles [26–35]. These alterations in part occur in conjunction with translocation of TF within the cell membrane [36]. However, the mechanism by which TF is restricted to the various regions has not been elucidated. These post-translational alterations may influence different TF-mediated processes. For example, phosphorylation of TF appears to be involved in the incorporation and release of TF within microvesicles [26] while de-palmitoylation affects TF activity on the cells [30]. However, de-palmitoylation appears to precede phosphorylation and suggests an order of events. A number of membrane-associated proteins are stabilised and restricted by interaction with MAGI proteins, which is mediated through the ww domains and the PDZ domains of MAGI proteins [13–19, 37–39]. Moreover, phosphorylation of proteins has been shown to interfere with the binding to the PDZ domains in MAGI proteins [38].

**Fig. 8:**
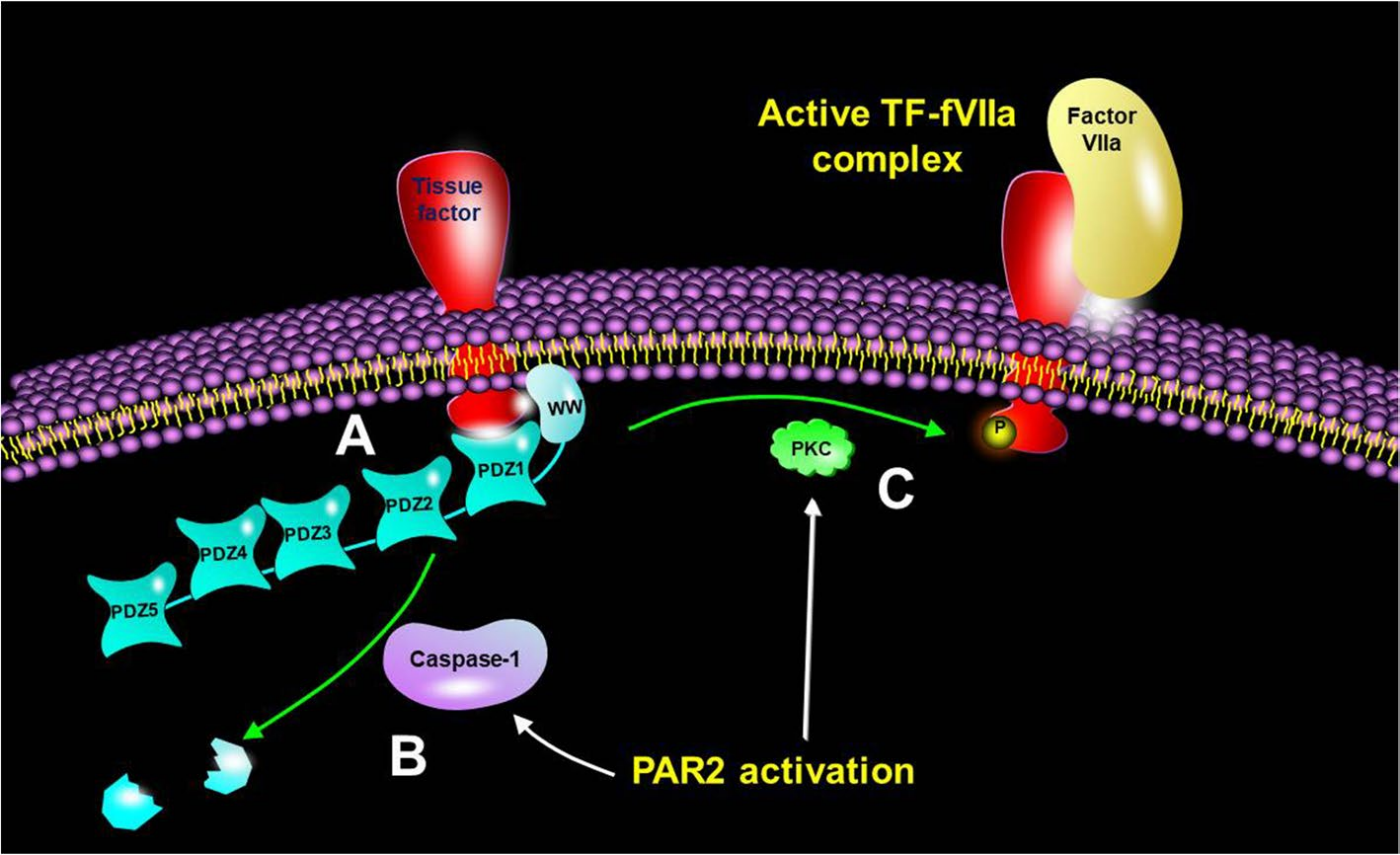
Schematic representation of the regulation of TF activity by interaction with MAGI1. **A** The interaction of the cytoplasmic domain of TF with the PDZ1 domain of MAGI1 restrains TF. Initiation of cell signalling following the activation of PAR2 on the cell surface may result in (**B**) the induction of capsase-1 leading to the degradation on MAGI1 into non-functional fragments. **C** Concurrent phosphorylation of TF by PKC at Ser253 prevents further binding with other MAGI1 proteins on the cells surface. These alterations collectively may promote the processing of TF permitting interaction with fVIIa as an active complex

In silico evaluation of TF sequence suggested the possibility of an interaction between the cytoplasmic domain of TF, specifically a putative PDZ-binding domain TF_256-260_ (ENSPL) and the PDZ domains in MAGI1, MAGI3 and MAGIX proteins. Examination of MDA-MB-231 cells using PLA indicated the likely association of TF mainly with MAGI1 (Fig. 1). This was further validated by both the co-immunoprecipitation of MAGI1 with TF, and also co-immunoprecipitation of TF with MAGI1 (Fig. 2). PAR2 activation in MDA-MB-231 cells resulted in reduced proximity between TF and MAGI1 that reached maximum dissociation at 20 min post-activation. Pull-down assays using peptides corresponding to the cytoplasmic domain of TF in different phosphorylation states indicated that MAGI1 preferentially interacted with non-phosphorylated and Ser258-phosphorylated TF, but the presence of the phosphate group associated with Ser253 hindered this interaction. In addition, lower ability of MAGI1 and MAGI3 to interact with Ser258-phosphorylated TF was detected which may arise from the interaction of the two ww domains within MAGI proteins with the phosphoserine-proline motif (termed an MPM-2 motif) within the cytoplasmic domain of TF [25, 40] and may be involved in the recycling of TF. We previously showed that the activation of PAR2 resulted in maximal phosphorylation of Ser253 at around 20 min in MDA-MB-231 cells [24, 26]. Since phosphorylation of protein domains is known to disrupt the interaction with PDZ domains, this may explain the dissociation of TF from MAGI1. In contrast, time-course pull down assay using the non-phosphorylated peptide also showed a drop in the amount of captured MAGI1. MAGI1 has been shown to be a target for caspase-1 [41] which is also known to promote the cell surface translocation of TF [42]. The expression of either the PDZ1 domain alone, or the N-terminal peptide of MAGI1 including the PDZ1 domain, resulted in increased thrombin generation potential which was directly dependent on the protease activity of TF-fVIIa (Figs. 5 and 6). To further support a cellular and physiological role for this mechanism, we have also shown that the release of TF through the expression of N-terminal peptide of MAGI1, resulted in pro-survival and proliferative signalling, leading to increased cell proliferation (Fig. 7). Importantly, this signal was directly dependent on the proteolytic activity of TF-fVIIa complex and was mediated by the activation of PAR2. While full-length MAGI1 localises at the sub-membrane region within cells, the cleavage of MAGI1 has been reported to produce fragments which no longer retain functional integrity [41]. The N-terminal cleavage product of MAGI1 is translocated to the cytosol and the C-terminal portion accumulates in the nucleus. This suggests a possible alternative mechanism and implicates alterations in the function of MAGI1, promoted by cell activation, as the cause of the protein dissociation (Fig. 8). Concurrent phosphorylation of TF by PKC at Ser253 may also prevent further binding of the released TF to other MAGI1 molecules on the cell surface. Analysis of the five PDZ domains of MAGI1 proteins, by expression in cells indicated an association of TF with PDZ1 and also the N-terminal MAGI1 peptide when containing PDZ1, suggesting a putative binding site. Significantly, the expression of PDZ1 alone, or within the N-terminal domain increased TF activity. This may be due to the ability of the expressed PDZ1-hybrid peptide to release TF, by competing with the native MAGI1 and therefore, acting similarly to the caspase 1-digested MAGI1 fragment.

## Conclusions

In conclusion, the perturbation of cells often occurs as a consequence of injury or the activation of cells and may result in the exposure of TF activity and the activation of coagulation. This study has shown that TF may interact with MAGI1, by an interaction mediated through the N-terminal PDZ1 domain of MAGI1 (Fig. 8). This association in turn can restrict TF activity. However, the activation of cells transiently disrupts this interaction resulting in the exposure of the latent TF as an active procoagulant protein. Consequently, the regulation of TF and its release appears to include multiple steps which contribute to the stringent regulation of TF activity.

### Supplementary Information

**Additional file 1.**

## Data Availability

No datasets were generated or analysed during the current study.

## Abbreviations

TF: Tissue factor
fVIIa/Xa: Factor VIIa/fXa
MAGI: Membrane-associated guanylate kinase-with inverted configuration
PAR2: Protease activated receptor 2
