# Supplementary material for: Regulation of tissue factor activity by interaction with the first PDZ domain of MAGI1
Source: Thromb J. 2024 Jan 17;22:12. doi: 10.1186/s12959-023-00580-6 (PMC10792917; doi:10.1186/s12959-023-00580-6)
Supplement: Supplementary file 1 — Additional file 1. [file 12959_2023_580_MOESM1_ESM.doc]

**Supplementary Table 1**

Restriction enzymes used:

Forward: BamHI

Reverse: HindIII

**N-terminal – excluding PDZ1**

Forward: CCCGGAGGATCCGATGTCCAAAGTGATCCAGAAGAAGAACCACT

Reverse: GAGGCCAAGCTTTTAAATGAACTTGCCTTTCAACTCAGAAGGG

**N-terminal – including PDZ1**

Forward: CCCGGAGGATCCGATGTCCAAAGTGATCCAGAAGAAGAACCACT

Reverse: GATAAAGCTTTTAGCAGAGTTCAAGGTCCACGCTGGC

PDZ1

Forward: caagGGATCCGATGcacacaaagctgcggaaaagcagtcg

Reverse: GATAAAGCTTTTAGCAGAGTTCAAGGTCCACGCTGGC

PDZ2

Forward: agaaGGATCCGATGactgttcatattgtcaaagggccaatgggc

Reverse: GCCCAAGCTTTTATTGCACCAACAATGTGACCTCACTTC

PDZ3

Forward: ccagGGATCCGATGgacatcttcctctggagaaaagagactg

Reverse: CCACAAGCTTTTACCGCACCGTGAGATTGACGTGGCCTTGC

PDZ4

Forward: TgcaGGATCCGATGgacgtggagatccggcgcggggagaacg

Reverse: CAGTAAGCTTTTAATCTTGCTCCTGTGCTGCTTGGGGC

PDZ5

Forward: agatGGATCCGATGactgtggaactggaaagaggagc

Reverse: CTCCAAGCTTTTACAGAAACAGACGAACTCTGCGGC

**Supplementary Table 2**

**PDZ Domain Name Domain Binding Site Sequence Peptide Sequence SVM Decision Score Ensembl Protein ID**

ARHGAP23-1 GFGFTLNGKNKGYSIS ENSPL 0.03 ENSP00000423381

GRIP2-5 ELGITISSSDKSMEVC SPLNV 0.056

LIN7B-1 GLGFNIMGSRIGHEVA KENSP 0.184

LIN7C-1 GLGFNIMGSRIGHEVA KENSP 0.184

**MAGI1-6 GFGFSLRGLRAGHSIG ENSPL 0.429 ENSP00000423381**

**MAGI3-6 GFGFSLRGLRAGHTIG ENSPL 0.438 ENSP00000423381**

**MAGIX-1 GFGLTLGGRGLGHAVG ENSPL 0.48 ENSP00000423381**

MPP4-1 FKGATIKRARIGPEIS KENSP 0.057

PDLIM1-1 GWGFRLVGSRTSHLQC ENSPL 0.035 ENSP00000423381

PDLIM5-1 PWGFRLQGSSKGHLQC ENSPL 0.073 ENSP00000423381

PDZK1-3 GYGFYLRGKDDSHDVG ENSPL 0.069 ENSP00000423381

PDZRN4-1 TLGFNIIGSKLGHEVA KENSP 0.082

**RHPN1-1 GFGLTLRGAAISHAVG ENSPL 0.603 ENSP00000423381**

SHROOM2-1 PWGFTLKGTKESRQIS KENSP 0.172

SLC9A3R1-2 GYGFNLHDRSDSHGVG ENSPL 0.28 ENSP00000423381

SLC9A3R2-2 GYGFNLHSRSDSHAVR ENSPL 0.195 ENSP00000423381

SNTA1-1 ALGISIKGSKFLHDVT KENSP 0.223

SNTB1-1 GLGISIKGSKFLHDVA KENSP 0.078

SNTB2-1 GLGISIKGSKFLHDVA KENSP 0.078

SNTG1-1 GFGLSIKGSKSQHEVA KENSP 0.384

SNTG2-1 GLGLSIKGSKFQHEVA KENSP 0.379

**Supplementary Figure 1**

**
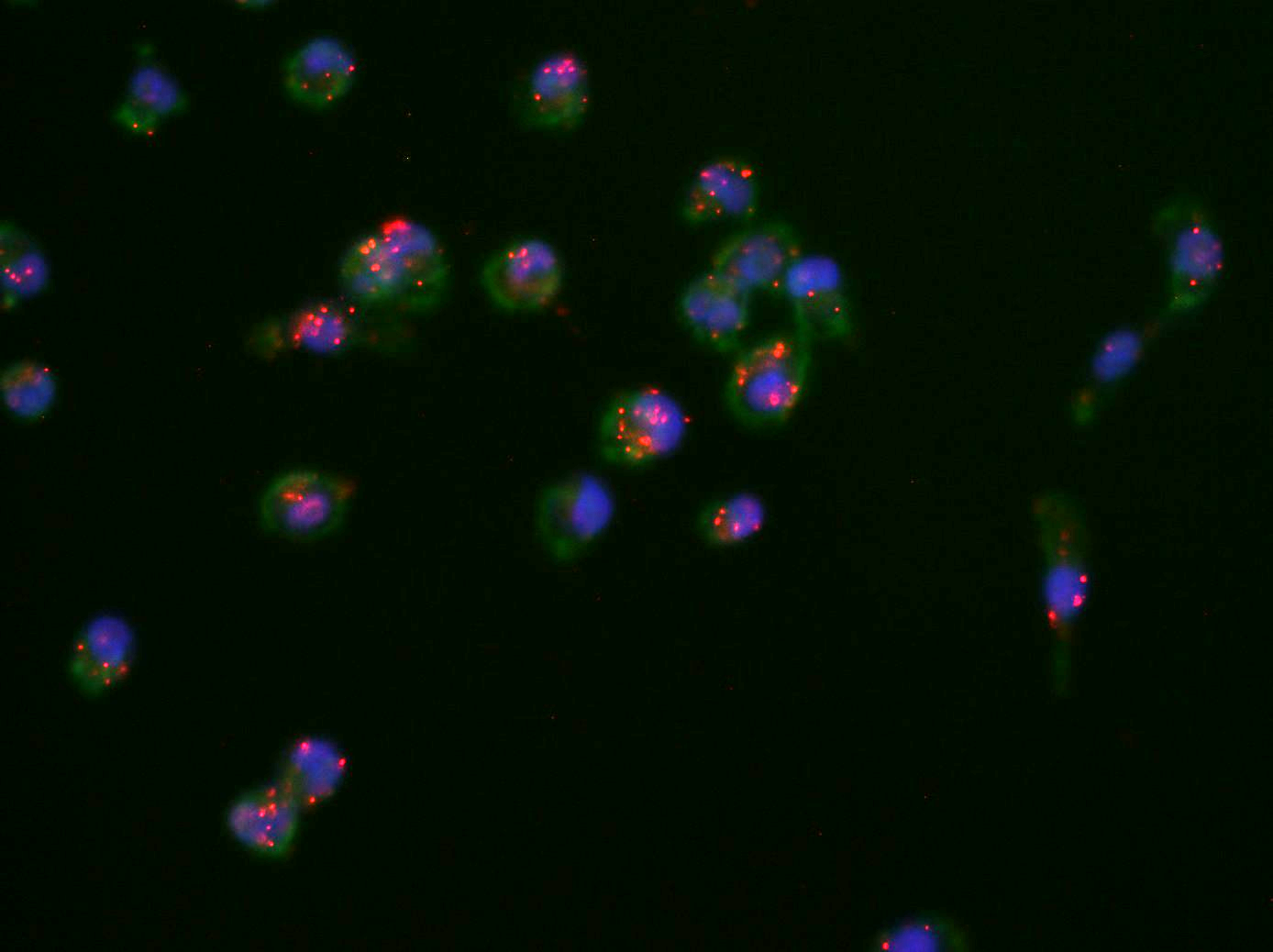

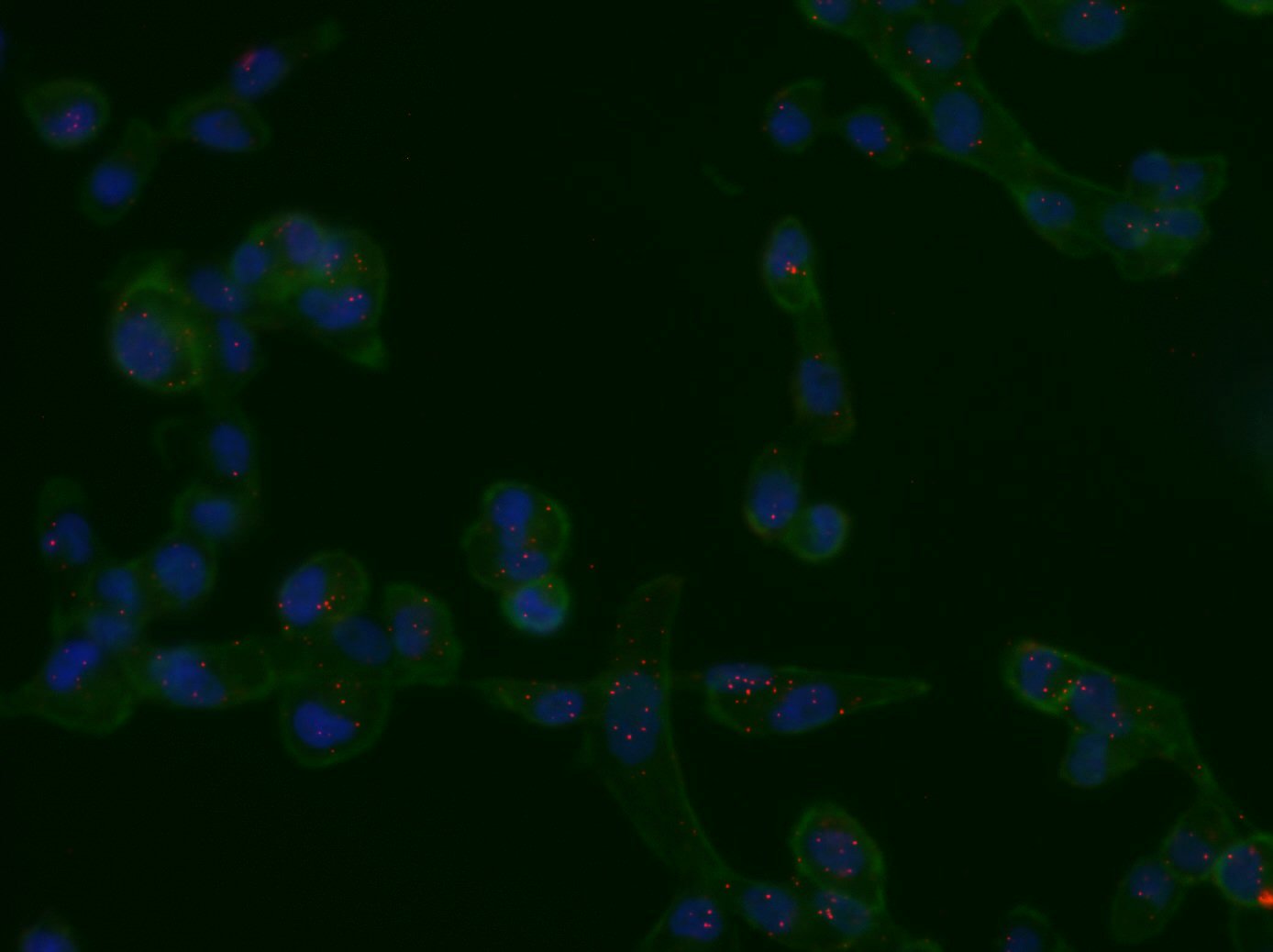
**

10 µm

MDA-MB-231 cells (10^3^) were seeded out into 35 mm-glass based μ-dishes and adapted to serum-free, washed and fixed with 4% (v/v) paraformaldehyde for 15 min. The cells were washed three times with PBS and permeabilised with Triton X-100 0.1% (v/v) in PBS, for 5 min. All samples were blocked with Duolink blocking buffer for 1 h and incubated overnight with combinations of antibodies as follows, at 4°C. The proximity between TF and MAGI1-3 were examined using a mouse anti-TF antibody HTF1 (5 μg/ml) together with A) a rabbit anti-TF antibody (FL295; 5 μg/ml) or B) an isotype IgG. The antibodies were diluted in the provided antibody diluent and blocked with the provided blocking buffer. The cells were washed three times with PBS and PLA performed according to the manufacturer’s instructions. The cells were labelled with DAPI (2 μg/ml) and Phalloidin-FITC (2 µg/ml). Images were acquired using a Zeiss Axio Vert.A1 inverted fluorescence microscope with a ×40 magnification. (RED= PLA incidences; GREEN = Phalloidin; BLUE = DAPI).

10 µm

**Supplementary Figure 2**


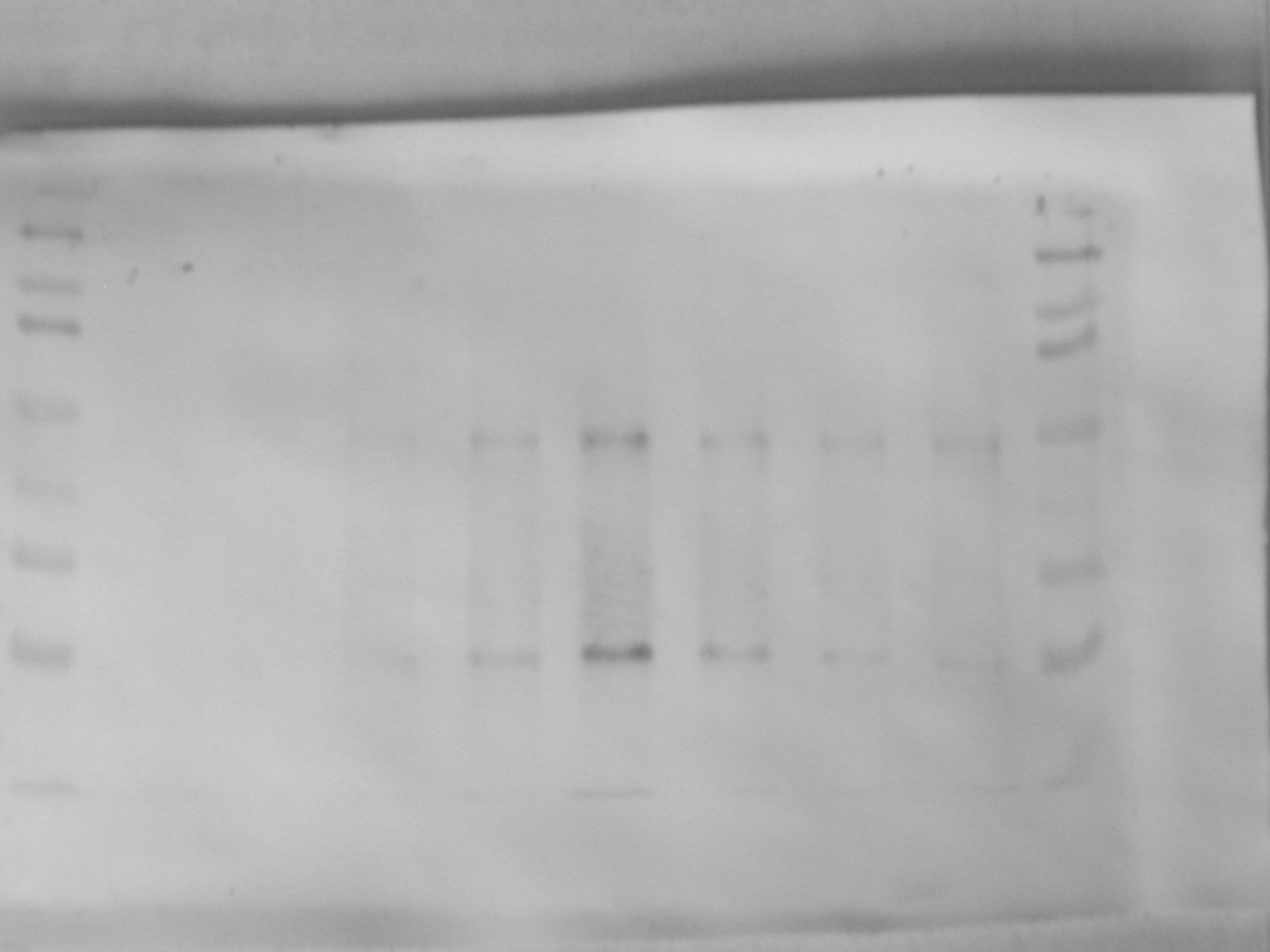


Markers Cell HTF-1 IgG No

lysate antibody Isotype antibody


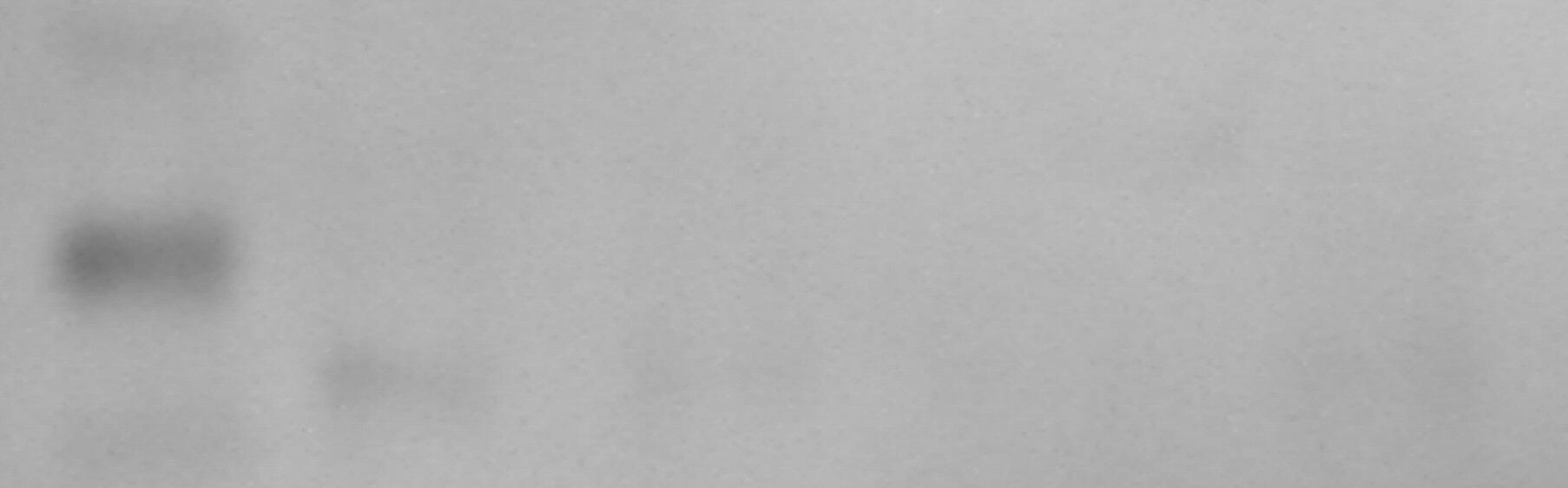


Markers Cell HTF-1 IgG No

lysate antibody Isotype antibody

140 kDa

70 kDa

50 kDa

Rhophilin-1


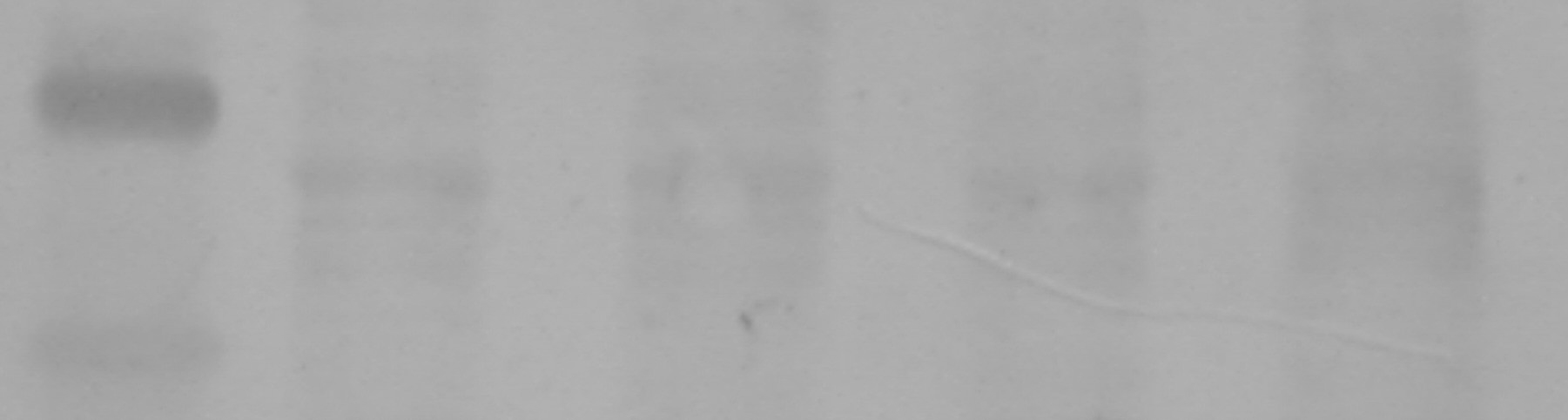


Markers No IgG HTF-1 Cell

antibody Isotype antibody lysate

250 kDa

140 kDa

70 kDa

MAGI2


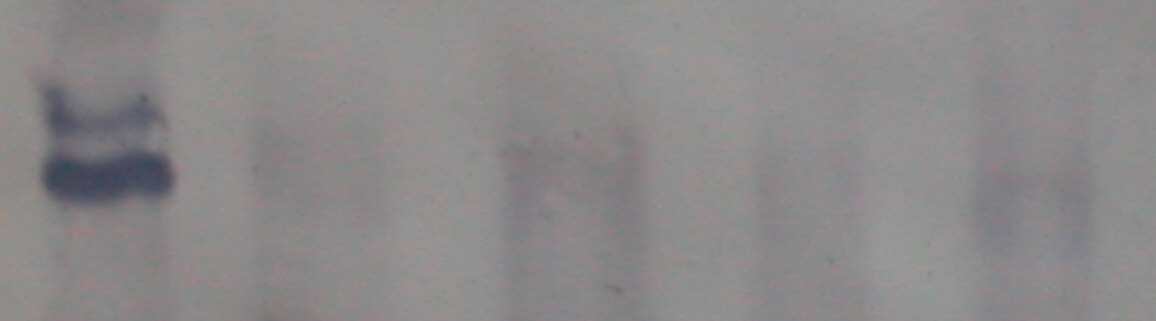


Markers No IgG HTF-1 Cell

antibody Isotype antibody lysate

140 kDa

100 kDa

70 kDa

MAGI3


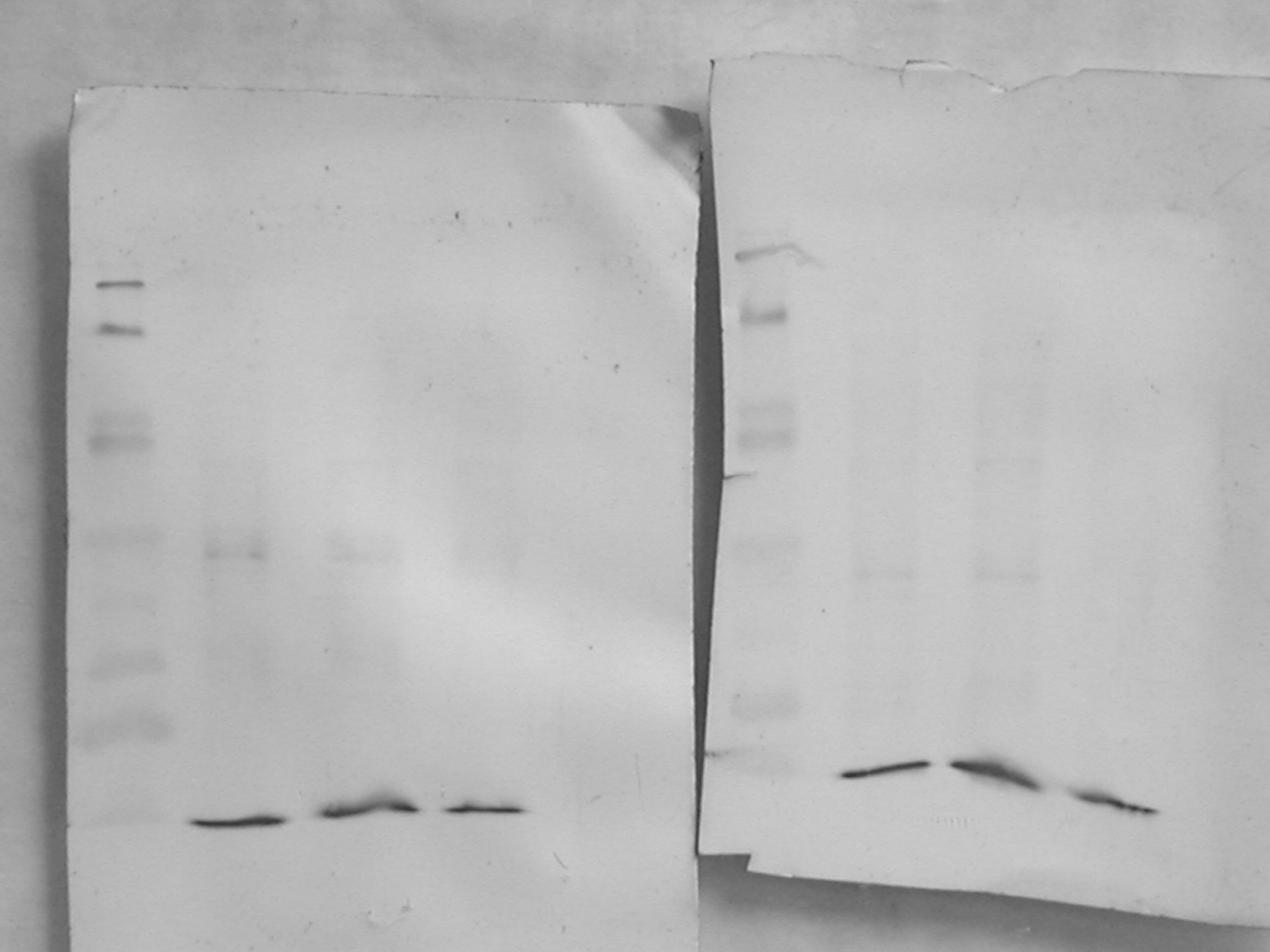


Markers Cell MAGI3 MAGI2 MAGIX

lysate antibody antibody antibody

70 kDa

50 kDa

40 kDa

30 KDa

Tissue factor

A)

70 kDa

50 kDa

30 kDa

MAGIX

B)

MDA-MB-231 cells were propagated in 25 cm^2^ flasks, washed with phosphate-buffered saline (PBS) pH 7.4. Cells were lysed in PhosphoSafe Extraction Reagent containing protease inhibitor cocktail and cell debris removed by centrifugation. A) TF was immunoprecipitated from cell lysates (2 × 10^5^) using the anti-TF antibody (HTF-1; 4 µg). To ensure specificity, a mouse IgG isotype (4 µg) was also included. All samples were incubated at 4°C overnight with gentle shaking. Pureproteome protein A-magnetic beads (10 µl) was added to all samples and controls and incubated at 4°C for 90 min. The samples were then placed in a magnetic stand and the supernatant removed, washed five times with PBST (1 ml) and the samples denatured in SDS-PAGE loading buffer (70 µl). All samples were diluted in Laemmli's buffer (1:1 v/v, except the cell lysates which were diluted 1:10 v/v with buffer first) and were separated by 12% (w/v) SDS-PAGE, transferred onto nitrocellulose membranes and then blocked with TBST (10 mM Tris-HCl pH 7.4, 150 mM NaCl, 0.05% Tween-20). The membranes were then probed using a polyclonal anti-MAGI2, anti-MAGI3 (46), anti-MAGIX (D-18) and anti-Rhophilin-1 (I-19) antibodies diluted 1:4000 (v/v) in TBST. The membranes were then washed three times with TBST and probed with a goat anti-mouse, goat-anti-rabbit, or donkey anti-goat alkaline phosphatase-conjugated antibodies as appropriate, diluted 1:4000 (v/v), and incubated for 90 min. TF bands were then visualised using the Western Blue stabilised alkaline phosphatase-substrate and recorded and analysed using ImageJ program. B) Immunoprecipitation was carried out using the anti-MAGI3, anti-MAGI2 or anti-MAGIX antibodies (4 µg). Western blot analysis was carried out as described above and the membranes were probed with anti-TF antibody (HTF-1) and developed using the goat anti-mouse alkaline phosphatase antibody, as described above.
